# Association between polymorphisms in long non-coding RNA *PRNCR1* in 8q24 and risk of colorectal cancer

**DOI:** 10.1186/1756-9966-32-104

**Published:** 2013-12-13

**Authors:** Lijuan Li, Ruifen Sun, Yundan Liang, Xinmin Pan, Zhaohui Li, Peng Bai, Xiaofeng Zeng, Dongxian Zhang, Lin Zhang, Linbo Gao

**Affiliations:** 1 Laboratory of Molecular and Translational Medicine, West China Institute of Women and Children’s Health; Key Laboratory of Obstetric & Gynecologic and Pediatric Diseases and Birth Defects of Ministry of Education, West China Second University Hospital, Sichuan University, Chengdu, Sichuan 610041, P.R. China; 2 Department of Forensic Biology, West China School of Preclinical and Forensic Medicine, Sichuan University, Chengdu, Sichuan 610041, P.R. China; 3 Central Laboratory, Yunnan University of Chinese Traditional Medicine, Kunming 650500, Yunnan, P.R. China; 4 Department of Forensic Pathology, College of Forensic Medicine, Henan University of Science and Technology, Luoyang, Henan 471003, P.R. China; 5 Secondary Department of General Surgery, Luoyang Central Hospital Affiliated to Zhengzhou University, Luoyang, Henan 471003, P.R. China; 6 Department of Forensic Medicine, Kunming Medical University, Kunming, Yunnan 650500, P.R. China

**Keywords:** Colorectal cancer, LncRNAs, Polymorphism

## Abstract

**Background:**

Genome-wide association studies have identified that genetic variants in 8q24 confer susceptibility to colorectal cancer (CRC). Recently, a novel lncRNA (*PRNCR1*) that located in the 8q24 was discovered. Single nucleotide polymorphisms (SNPs) in the lncRNAs may influence the process of splicing and stability of mRNA conformation, resulting in the modification of its interacting partners. We hypothesized that SNPs in the lncRNA *PRNCR1* may be related to the risk of CRC.

**Methods:**

We conducted a case–control study and genotyped five tag SNPs in the lncRNA *PRNCR1* in 908 subjects including 313 cases with CRC and 595 control subjects using polymerase chain reaction–restriction fragment length polymorphism (PCR-RFLP) assay.

**Results:**

In overall analyses, we found that the rs13252298 and rs1456315 were associated with significantly decreased risks of CRC. In stratification analyses, we found that CRC patients carrying the rs1456315G were likely to have a tumor size of greater than 5 cm (G vs. A: adjusted OR = 1.56, 95% CI: 1.10-2.23). Additionally, patients with the rs7007694C and rs16901946G had decreased risks to develop poorly differentiated CRC, whereas patients with the rs1456315G had an increased risk to develop poorly differentiated CRC.

**Conclusion:**

These findings suggest that SNPs in the lncRNA P*RNCR1* may contribute to susceptibility to CRC.

## Introduction

Cancer including colorectal cancer (CRC) is a disease accumulated with multistep genetic and epigenetic level changes and with a complex etiology [1]. Genome-wide association scans and subsequent observational replication studies have identified that genetic variants located at the chromosomal region 8q24 confer susceptibility to CRC [2-17]. However, the region was called “gene-desert” area because it does not harbor any candidate gene except for the putative gene *POU5F1P1* whose function is unknown [18], causing the function of the variations in the susceptibility loci is not well established.

Recently, a ~13 kb long non-coding RNAs (lncRNA) was discovered that was transcribed from the “gene-desert” region of chromosome 8q24 (128.14-128.28 Mb) [19]. The lncRNA, termed prostate cancer non-coding RNA 1 (*PRNCR1*), was reported to be involved in the carcinogenesis of prostate cancer [19]. Therefore, further characterization of lncRNA related single nucleotide polymorphisms (SNPs) may open a new avenue for functional analysis of cancer susceptibility loci identified by genome-wide association study, especially when it was located in introns or “gene-desert” region.

LncRNAs are RNA polymerase II-transcribed, polyadenylated, and frequently alternatively spliced RNAs [20,21] with the features of cell-type specific expression patterns [22-24], distinct subcellular localizations [24], linkage to various diseases [25], and evolutionary selection of the lncRNA sequence [26,27]. LncRNAs can be intergenic, intronic, antisense or overlapping with protein-coding genes or other ncRNAs [26,28-30]. Recent studies have revealed the contribution of ncRNAs as proto-oncogene [31], tumor suppressor gene [32], drivers of metastasis transformation in cancer development [33]. The expression of lncRNAs is deregulated in different cancers, including colon cancer [34]. Several lines of evidence have shown that SNPs in lncRNAs may influence the process of splicing and stability of mRNA conformation, resulting in the modification of its interacting partners [19,35].

In this study, we hypothesized that SNPs in lncRNAs may be involved in the risk of CRC. To test this hypothesis, we selected five tag SNPs in the lncRNA *PRNCR1* in the “gene-desert” region of 8q24 (i.e., rs1016343, rs13252298, rs7007694, rs16901946, and rs1456315), and genotyped the SNPs in a case–control study of 313 cases with CRC and 595 ethnicity-matched controls in a Chinese population.

## Subjects and methods

### Subjects

Totally, 908 subjects attended our case–control study comprising 313 cases (313 patients with CRC including 199 males and 114 females) and 595 control subjects (289 males and 306 females). Diagnosis of CRC was confirmed by histopathological examination and those who had inflammatory bowel disease were excluded. Patients were recruited from the Luoyang Central Hospital and the West China Hospital, Sichuan University between January 2010 and February 2012. Control subjects including 595 healthy volunteers who came to the West China Hospital just for routine check-up during the same time as the patients. Individuals were excluded if there was any evidence of personal or family history of cancer or inflammatory diseases in the intestine, such as ulcerative colitis or Crohn’s colitis. There was no significant difference between patients and control subjects in terms of ethnicity distribution. Written informed consent was obtained from all subjects attending this study, and the study was performed with the approval of the ethics committee of the hospital.

### Selection of SNPs

We searched tag SNPs in the lncRNAs *PRNCR1* in the chromosomal region 8q24 using UCSC (http://genome.ucsc.edu/) with the selection criteria of the minor allele frequency more than 0.10 in Asians. Finally, five tag SNPs were identified: rs1016343 (Chr8-128162479), rs13252298 (Chr8-128164338), rs7007694 (Chr8-128168348), rs16901946 (Chr8- 128170107), and rs1456315 (Chr8-128173119).

### Genotyping

2 mL peripheral blood used for genotyping assay was obtained from each subject after their admission to the hospital, and each subject was interviewed to obtain demographic and clinical information. Genomic DNA was extracted from the blood of the subjects using a commercial extraction kit (Bioteke Corporation, Beijing, China) according to the manufacturer’s directions. We used a polymerase chain reaction–restriction fragment length polymorphism (PCR-RFLP) assay to acquire all the genotypes of the five SNPs (i.e., rs1016343, rs13252298, rs7007694, rs16901946, and rs1456315). Primer sequences, reaction conditions, restriction enzymes (New England BioLabs Inc; Beverly, MA, USA.) and length of polymerase chain reaction products are summarized in Additional file 1: Table S1. Restriction fragments were distinguished on 6% polyacrylamide gels and visualized by silver staining to identify the genotypes. For quality control, the PCR products of the five SNPs with different genotypes were confirmed by DNA sequencing, and the results were 100% concordant. Additionally, 10% of the whole samples were repeated, and positive and negative controls were used in each 96-well PCR plate.

### Statistical analysis

Allele and genotype frequencies of the five SNPs were obtained using Modified-Powerstates standard edition software. Hardy-Weinberg equilibrium was tested with a goodness of fit chi-square test (with one degree of freedom) to compare the observed genotype frequencies among the subjects with the expected genotype frequencies. The demographic and clinical data of the two groups were compared using the chi-square test. Bivariate logistic regression was used to calculate the odds ratios (ORs), 95% confidence intervals (CIs), and corresponding *p* values after adjustment for age and gender. *P* < 0.05 is considered statistically significant. All data were analyzed using the SPSS for Windows software package version 13.0 (SPSS Inc., Chicago. IL).

## Results

The five SNPs of rs1016343, rs13252298, rs7007694, rs16901946, and rs1456315 in 8q24 were successfully genotyped for 908 subjects. The clinical features of subjects enrolled in our study are shown in Table 1. The genotype frequencies of the five polymorphisms in the control group met the requirements of the Hardy-Weinberg equilibrium (*P* >0.05). The genotype and allele frequencies of the five SNPs are summarized in Table 2. The AG genotype and G allele of rs13252298 were associated with a significantly decreased risk of CRC, compared with the AA genotype and A allele (AG vs. AA, adjusted OR = 0.67, 95% CI: 0.49-0.91, *p* = 0.01; G vs. A, adjusted OR = 0.75, 95% CI: 0.60-0.94, *p* = 0.01, respectively). Moreover, the AG genotype of rs1456315 was also associated with a significantly decreased risk of CRC, compared with the AA genotype (AG vs. AA, adjusted OR = 0.66, 95% CI: 0.48-0.90, *p* = 0.01). However, no significant association was observed between the other SNPs and risk of CRC. Besides, we examined the linkage disequilibrium (LD) plot,and the 5 SNPs was not in LD (data not shown).

**Table 1:** Demographics of patients with CRC and controls

**Variables**	**Controls n = 595 (%)**	**CRC n = 313 (%)**
Mean age (y)	51.5(±10.9)	59.8(±13.8)
Gender		
Male	289(48.6)	199(63.6)
Female	306(51.4)	114(36.4)
Tumor size		
<5 cm		174(55.6)
≥5 cm		139(44.4)
Differentiated status		
Well-Moderately		242(77.3)
Poorly-Undifferentiated		71(22.7)
Clinical stage		
I-II		168(53.7)
III- IV		145(46.3)
Metastasis		
Yes		141(45.0)
No		172(55.0)

**Table 2:** Genotype and allele frequencies of the five SNPs between cases and controls

**Polymorphisms**	**Controls (n = 595) (%)**	**CRC (n = 313) (%)**	**Adjusted OR (95% CI)**	***p***
rs1016343				
CC	227(38.1)	117(37.4)	1.0(ref)	
CT	276(46.4)	156(49.8)	1.33(0.82-2.14)	0.25
TT	92(15.5)	40(12.8)	1.13(0.83-1.55)	0.44
C	730(61.3)	390(62.3)	1.0(ref)	
T	460(38.7)	236(37.7)	1.14(0. 92–1.41)	0.24
rs13252298				
AA	264(44.4)	166(53.0)	1.0(ref)	
AG	270(45.4)	121(38.7)	0.67(0.49-0.91)	0.01
GG	61(10.2)	26(8.3)	0.64(0.38-1.09)	0.10
A	798(67.1)	453(72.4)	1.0(ref)	
G	392(32.9)	173(27.6)	0.75(0.60-0.94)	0.01
rs7007694				
TT	362(60.8)	184(58.8)	1.0(ref)	
CT	208(35.0)	107(34.2)	1.04(0.76-1.42)	0.80
CC	25(4.2)	22(7.0)	1.60(0.85-3.03)	0.15
T	932(78.3)	475(75.9)	1.0(ref)	
C	258(21.7)	151(24.1)	1.15(0.90-1.46)	0.27
rs16901946				
AA	338(56.8)	175(55.9)	1.0(ref)	
AG	232(39.0)	117(37.4)	0.96(0.71-1.31)	0.80
AG/GG	257(43.2)	138(44.1)	1.03(0.77-1.38)	0.85
A	908(76.3)	467(74.6)	1.0(ref)	
G	282(23.7)	159(25.4)	1.10(0.86-1.39)	0.45
rs1456315				
AA	294(49.4)	167(53.4)	1.0(ref)	
AG	262(44.0)	119(38.0)	0.66(0.48-0.90)	0.01
GG	39(6.6)	27(8.6)	1.09(0.62-1.91)	0.78
A	850(71.4)	453(72.4)	1.0(ref)	
G	340(28.6)	173(27.6)	0.86(0.70-1.08)	0.18

OR: odds ratio; CI: confidence interval; Ref: reference.

When patients were divided according to tumor size, differentiated status, clinical stage, and metastasis status, we found that CRC patients carrying the rs1456315G allele were likely to have a tumor size of greater than 5 cm (G vs. A: adjusted OR = 1.56, 95% CI: 1.10-2.23). Additionally, patients with the rs7007694C allele and rs16901946G allele had a decreased risk to develop poorly differentiated CRC (rs7007694 C vs. T: adjusted OR = 0.46, 95% CI: 0.28-0.77; rs16901946 G vs. A: adjusted OR = 0.59, 95% CI: 0.37-0.94, respectively). Interestingly, patients with the rs1456315G allele had an increased risk to develop poorly differentiated CRC (adjusted OR = 1.54, 95% CI: 1.03-2.31) (Table 3).

**Table 3:** **Stratified analyses of lncRNA *PRNCR1 *** **polymorphisms with clinical features in patients with CRC (minor allele vs. major allele)**

**Polymorphisms**	**Adjusted OR for age and gender (95% CI)/*p***			
	**Tumor size (≥5 cm)**	**Differentiated status (poorly)**	**Clinical stage (III-IV)**	**Metastasis (yes)**
rs1016343C/T	0.82(0.59-1.13)/0.22	1.05(0.72-1.55)/0.79	1.07(0.77-1.49)/0.70	1.27(0.91-1.78)/0.16
rs13252298A/G	1.07(0.75-1.52)/0.72	1.21(0.80-1.82)/0.37	0.85(0.59-1.21)/0.36	0.76(0.53-1.10)/0.15
rs7007694T/C	0.74(0.51-1.08)/0.11	0.46(0.28-0.77)/0.003	1.04(0.71-1.51)/0.85	1.11(0.76-1.62)/0.59
rs16901946A/G	0.84(0.59-1.22)/0.36	0.59(0.37-0.94)/0.03	1.09(0.76-1.58)/0.64	1.26(0.87-1.83)/0.22
rs1456315A/G	1.56(1.10-2.23)/0.01	1.54(1.03-2.31)/0.04	1.16(0.81-1.66)/0.43	1.06(0.73-1.52)/0.77

CRC: colorectal cancer; OR: odds ratio; CI: confidence interval.

The smaller size, well differentiated status, clinical stage I-II, and the ones without metastasis were made as references, respectively.

## Discussion

In the present study, for the first time, we provided evidence that SNPs (i.e., rs13252298, rs7007694, rs16901946, and rs1456315) in the lncRNA *PRNCR1* at the “gene-desert” region in 8q24 might be associated with CRC susceptibility. We identified the rs13252298 and rs1456315 were associated with significantly decreased risks of CRC. In stratification analyses, we found that the rs1456315 was related to the tumor size of CRC. Moreover, patients with the rs7007694C allele and rs16901946G allele had decreased risks to develop poorly differentiated CRC, whereas patients with the rs1456315G allele had an increased risk to develop poorly differentiated CRC. These findings indicate that the polymorphisms in the lncRNA *PRNCR1* may be related to the development of CRC, offering a novel and potential strategy for functional analysis of susceptibility loci to human diseases.

It has been shown that lncRNAs have developmental and tissue specific expression patterns, with an aberrant regulation in various diseases, including cancer [24,36-44]. LncRNAs have been reported to be involved in cancer development in three different ways: Firstly, some lncRNAs take part in the process as oncogene or oncogene regulator, for example, *MALAT1* gene in non-small cell lung cancer [45] and *H19* in colon cancer [46]. The expression of *MALAT1* was up-regulated in many kinds of human cancers such as breast cancer, prostate cancer, colon cancer, liver cancer, and uterus cancer [44,47-49]. Mice lacking *H19* presented an increased polyp count which is related to CRC [50]. Secondly, lncRNA may be related to cancer metastasis or prognosis. Gupta et al. reported a lncRNA *HOTAIR* which was associated with cancer metastasis and poor survival [33]. Thirdly, lncRNAs appear as tumor suppressor gene: *MEG3* is the first lncRNA proposed to function as a tumor suppressor and also a top level regulatory RNA because of its ability stimulating both p53-dependent and p53-independent pathways [32,51]. Recurring chromosomal aberrations can influence the expression of many lncRNAs, such as disrupted in schizophrenia 1 and 2 (*DISC1* and *DISC2*), which were involved in the development of various diseases [52,53]. For instance, a large number of SNPs in the *DISC1* genomic sequence have been reported to be associated with schizophrenia spectrum disorder [54,55].

Emerging evidence has demonstrated that SNPs located in non-coding regions may be used as susceptibility factors to several diseases. Scott et al. reported that SNPs adjacent to the lncRNA *ANRIL* were associated with increased risks of type 2 diabetes [56]. The viewpoint was also confirmed by a separate study, which reported that distinct SNPs in the lncRNA *ANRIL* locus were associated with susceptibility to coronary artery disease and atherosclerosis [57]. Further characterization of the identified polymorphisms showed that SNPs can disrupt *ANRIL* splicing, leading to a circular transcript that is resistant to RNase digestion [35]. The circularized transcripts have effect on *ANRIL* normal function and influence *INK4*/*ARF* expression. Other evidence is from the recent study of leukemia and CRC which identified both germline and somatic mutations in lncRNA genes [58]. Recently, a novel lncRNA, named *PRNCR1*, has been discovered and was reported to be up-regulated in prostate cancer [19]. SNPs in the lncRNA *PRNCR1* gene might influence the predicted secondary structure of *PRNCR1* mRNA, alter the stability of the lncRNA *PRNCR1* or the mRNA conformation, and result in the modification of its interacting partners [19]. Since the lncRNA *PRNCR1* located in 8q24 which was a susceptibility locus to CRC [2-17,59], we hypothesized that SNPs in this region may have roles in the development of CRC. Our findings confirmed our hypothesis. We found that tag SNPs in the lncRNA *PRNCR1* may be a protective factor against CRC, suggesting that SNPs in lncRNA may be involved in the tumorigenesis of CRC. Although Chung and colleague’s report suggested that the lncRNA *PRNCR1* was associated with prostate carcinogenesis and may play a role through the regulation of androgen receptor (AR) transactivation activity [19], no report investigated the relationship between the region 2 of 8q24 and CRC risk. Moreover, there were reports suggested that AR also participated in the pathologic process of CRC through TGFβ pathway [60,61]. In this study, we found that the rs1456315 was also associated with clinical features of CRC, which was consistent with the report by Chung et al. [19].

Although we detected the association between SNPs in lncRNA and CRC, there were limitations needed to be mentioned in our study. One is that the follow-up information is blank, which limited our further analysis on the association between SNPs in lncRNA and CRC prognosis. Another is that the study subjects are all ethnic Han Chinese, and the sample size is moderate. Further large-scale studies in different populations, therefore, still need to be done.

## Conclusion

In conclusion, we found that the variant genotypes of rs13252298 and rs1456315 may contribute to a decreased risk of CRC. Moreover, the rs7007694, rs16901946, and rs1456315 polymorphisms were associated with the tumor size and differentiated status of patients. Association studies with diverse populations and further functional analysis of the variants are needed to verify our findings. Once our understanding of lncRNAs language is clear, we will be able to classify diseases based on the identified mutations and their effect on lncRNA function.

## Competing interests

None of the authors has any potential financial conflict of interest related to this manuscript.

## Authors’ contributions

LLJ, GLB and ZL designed the study. LLJ, SRF, LYD, PXM, LZH, BP, ZXF and ZhDX performed genotyping. LLJ, SRF and GLB conducted statistical analysis. LLJ and ZL wrote the manuscript. All authors read and approved the final manuscript.

## Supplementary Material

Additional file 1: Table S1 Primer sequences and reaction conditions for genotyping the five SNPs. Click here for file
