# Supplementary material for: Association between polymorphisms in long non-coding RNA PRNCR1 in 8q24 and risk of colorectal cancer
Source: J Exp Clin Cancer Res. 2013 Dec 13;32(1):104. doi: 10.1186/1756-9966-32-104 (PMC4029281; doi:10.1186/1756-9966-32-104)
Supplement: Additional file 1: Table S1 — Primer sequences and reaction conditions for genotyping the five SNPs. [file 1756-9966-32-104-S1.doc]

Supplementary table 1: Primer sequences and reaction conditions for genotyping the five SNPs.

| *db SNP ID* | *position* | *Primer sequence* | *Annealing temperature (℃)* | *Restriction enzyme* | *Product size (bp)* |
| --- | --- | --- | --- | --- | --- |
| rs1016343 | Chr8- [128162479](http://www.ncbi.nlm.nih.gov/sites/nuccore/NC_000008.9?report=graph&v=128161979:128162979&content=5&m=128162479!&mn=rs1016343&dispmax=1&currpage=1) | 5’-TCTCAATGCCCAAGAGATGAGC-3’  5’-GAACTGTGAAGCTGTGAGTAACCATG-3’ | 59 | *Nco* I | T:111  C:85+26 |
| rs13252298 | Chr8- [128164338](http://www.ncbi.nlm.nih.gov/sites/nuccore/NC_000008.9?report=graph&v=128163838:128164838&content=5&m=128164338!&mn=rs13252298&dispmax=1&currpage=1) | 5’-GCACTTGCTGTCTTCTCAGATACGAT-3’  5’-GCATTTCCTGAATTCCCAAAAGC-3’ | 59 | *EcoR* V | G:249  A:223+26 |
| rs7007694 | Chr8- [128168348](http://www.ncbi.nlm.nih.gov/sites/nuccore/NC_000008.9?report=graph&v=128167848:128168848&content=5&m=128168348!&mn=rs7007694&dispmax=1&currpage=1) | 5’-CGAATGCCATTTGTTTGGACG-3’  5’-CTCCACCTCCCAAAGAAGCTCC-3’ | 59 | *BSTU* I | T:133  C:112+21 |
| rs16901946 | Chr8- [128170107](http://www.ncbi.nlm.nih.gov/sites/nuccore/NC_000008.9?report=graph&v=128169607:128170607&content=5&m=128170107!&mn=rs16901946&dispmax=1&currpage=1) | 5’-CTCTTCCTTCTCTCACCCTTCTTTCC-3’  5’-TGGCAGAGGACTTTCTTTGAAAGC-3’ | 57 | *Msp* I | A:165  G:140+25 |
| rs1456315 | Chr8- [128173119](http://www.ncbi.nlm.nih.gov/sites/nuccore/NC_000008.9?report=graph&v=128172619:128173619&content=5&m=128173119!&mn=rs1456315&dispmax=1&currpage=1) | 5’-TGACAAGGGATTCCAAATAATC-3’  5’-AAGAACTGAGGTTGCTAATAAATGTA-3’ | 54 | *Rsa* I | A:143  G:118+25 |
